# Plasmonic Multi‐Layered Built‐in Hotspots Nanogaps for Effectively Activating Analytes

**DOI:** 10.1002/advs.202306125

**Published:** 2023-12-03

**Authors:** Lei Jiang, Xiaoyuan Wang, Jingyi Zhou, Qianqian Fu, Bihu Lv, Yixuan Sun, Liping Song, Youju Huang

**Affiliations:** ^1^ College of Material Chemistry and Chemical Engineering Key Laboratory of Organosilicon Chemistry and Material Technology Ministry of Education Hangzhou Normal University Hangzhou Zhejiang 311121 China; ^2^ Department of Scientific Facilities Development and Management Zhejiang Laboratory Hangzhou 311100 China; ^3^ Laboratory for Functional Molecules Materials Westlake University Hangzhou Zhejiang 310030 China

**Keywords:** activating analytes, built‐in nanogaps, multi‐layered nanostructures, near‐filed enhancement, surface‐enhanced Raman spectroscopy

## Abstract

Multi‐layered plasmonic nanostructures are able to highly promote the near‐field confinement and effectively activate analytes, which are of predominate significance but are extremely challenging. Herein, the semi‐open Au core@carved AuAg multi‐shell superstructure nanoparticles (multi‐Au@Ag‐Au NPs, multi = mono, bi, tri, tetra, and penta) are reported with a high designability on electromagnetic field and capability of effectively capturing analytes. By controlling synthetic parameters such as the number of galvanic exchange and Ag growth, multi‐Au@Ag‐Au NPs are successfully obtained, with tunable layer numbers and asymmetric nanoholes. Due to collective plasmon oscillations of multi‐layered built‐in nanogaps, the electromagnetic field strength of a single penta‐Au@Ag‐Au entity reach 48841. More importantly, the penta‐Au@Ag‐Au NPs show a remarkable light‐harvesting capability, which is adaptive to different Raman lasers, supporting high‐diversity detection. Additionally, the structural specificity allows analytes to be sufficiently captured into interior hotspots, and further achieve highly sensitive detection with limit of detection down to 3.22 × 10^−12^ M. This study not only provides an effective pathway for integrating abundant hotspots and activating target molecules in single plasmonic superstructure, but stimulates advancements in SERS substrates for various applications.

## Graphical abstract

The semi‐open multi‐Au@Ag‐Au  nanoparticles (NPs) show a remarkable capability of near‐field focusing and light confinement due to collective plasmon oscillations of multi‐layered built‐in nanogaps. The structural specificity allows analytes to be captured into hotspots center, enabling effective activation of surface‐enhanced Raman scattering (SERS) signals.

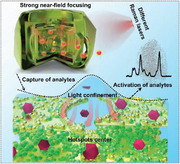

## 1 Introduction

Plasmonic metal nanomaterials (such as Au, and Ag) have attracted considerable research attention due to their unique optical, electronic, and magnetic properties, especially tunable localized surface plasmon resonances (LSPR).^[^ ^1^, ^2^, ^3^ ^]^ Originating from the collective oscillation of conduction electrons on metallic surfaces with resonant light, LSPR property allows for spatially confining incident light energy into specific regions of plasmonic nanomaterials where an enhanced and localized electromagnetic (EM) field (known as hotspots) can be formed.^[^ ^4^, ^5^, ^6^ ^]^ In particular, plasmonic nanomaterials are extensively used in surface‐enhanced Raman spectroscopy (SERS), a powerful technique being exploited widely in reaction monitoring, biomedical diagnostics and chemical analysis, *etc*.^[^ ^7^, ^8^, ^9^ ^]^ To enhance the light confinement and near‐field coupling in SERS‐active substrates, there has been in‐depth development of approaches for designing electromagnetic hotspots and electronic structure of nanomaterials.^[^ ^10^ ^]^

Generally, hotspots are enhanced on sharp tips due to the “lightning rod effect” and become remarkedly boosted once narrow gaps are formed among inter or intra‐nanostructures.^[^ ^11^, ^12^, ^13^ ^]^ Specifically, internally coupled plasmonic nanostructures with a built‐in dielectric nanogap are excellent candidates for hotspots engineering and electromagnetic field focusing.^[^ ^14^, ^15^ ^]^ In this regard, effective light‐matter interaction in subnanometer gaps can be induced owing to the strong EM field localized at their exterior and interior nanogaps. Although many strategies to fabricate plasmonic nanostructures with interior nanogap have been reported, most of them were confined to form either single‐layer or enclosed core‐shell nanostructures,^[^ ^16^, ^17^ ^]^ which hindered sufficient utilization of inner gaps. Therefore, it is highly advantageous to develop plasmonic substrates with multiple intra‐nanogaps and open channel for further improving plasmonic performance.

Multi‐layered hollow nanostructures have exhibited great promise for large near‐field enhancement owing to the presence of built‐in hot spots of high density.^[^ ^18^, ^19^ ^]^ For example, Park's group developed a broad library of plasmonic nanoframes consisting of double‐layered hollow open voids and thin metal ridges via multiple chemical steps, showing improved LSPR sensing sensitivity.^[^ ^20^, ^21^ ^]^ However, symmetric nanoframes were usually generated through complete etching of the certain facets, compromising the tunability of plasmonic properties and light‐harvesting capability. Besides, in terms of molecules without functional groups which are always in constant motion due to the weak affinity to metal surface,^[^ ^22^, ^23^, ^24^ ^]^ it remains challenging to effectively capture them into interior nanogaps of such symmetric hollow nanoframes. Therefore, the demand for high designability on plasmonic nanostructure which can not only enhance near‐field focusing but effectively activate all analytes remains open to be fulfilled.

**Figure 1:**
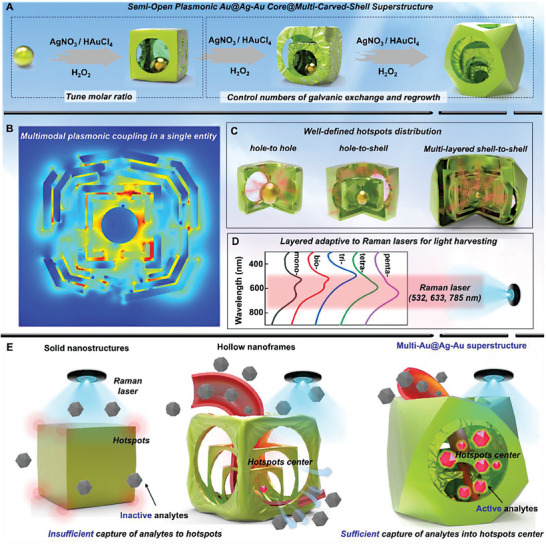
Schematic of the working principle of plasmonic Au core@multi‐carved‐shell superstructure. A) The synthesis process of the superstructure via sequential galvanic exchange and co‐deposition. B) The multimodal plasmonic coupling in a single penta‐Au@Ag‐Au superstructure. C) Hotspots distribution among carved nanoholes and different metallic layers as well as the coupling between the nanohole and adjecent layers. D) Penta‐Au@Ag‐Au NPs with a wide LSPR band were adaptive to Raman lasers for light harvesting. E) The comparison of the ability of capturing analytes into hotspots between traditional substrates (solid nanostructures and open nanoframes) and the proposed superstructures.

Herein, we report a class of semi‐open Au@Ag‐Au core@multi‐carved‐shell superstructure nanoparticles (multi‐Au@Ag‐Au NPs, multi = mono, bi, tri, tetra, and penta) to highly improve plasmonic performance enabled by tunable multi‐layered built‐in nanogaps and nanoholes. As illustrated in **Figure** **1**, using spherical Au nanoparticle (AuNP) as a core, multi‐Au@Ag‐Au NPs was fabricated through controlling the total number of galvanic replacement reaction and Ag growth, as well as the molar ratio of HAuCl_4_/AgNO_3_. The well‐tuned semi‐open multi‐Au@Ag‐Au NPs with different layer numbers and asymmetric nanoholes allowed systematic investigation of the corresponding plasmonic properties in detail. Remarkedly, due to multimodal plasmonic coupling effect from built‐in nanogaps distributed among carved nanoholes and different metallic layers, penta‐Au@Ag‐Au NPs exhibited ultrahigh electromagnetic field strength and enhancement factor. Owing to joint contribution of multi‐layered superstructures, the highly uniform penta‐Au@Ag‐Au NPs with wide LSPR absorption band were adaptive to different Raman lasers. Furthermore, the superior structure of penta‐Au@Ag‐Au NPs made it feasible to sufficiently capture target molecules into interior nanogaps with the assistance of interfacial assembly technology, thus allowing effective activation of SERS signals. We thus demonstrated highly quantitative and sensitive detection of different molecules even without functional groups via the proposed plasmonic substrate. This paves a new way for designing plasmonic nanostructures with well‐defined SERS hotspots that capture molecules actively, promising for widespread applications in biological, medical, and environmental fields.

## 2 Results and Discussion

### 2.1 Synthesis and Characterization of Multi‐Layered Superstructures

**Figure 2:**
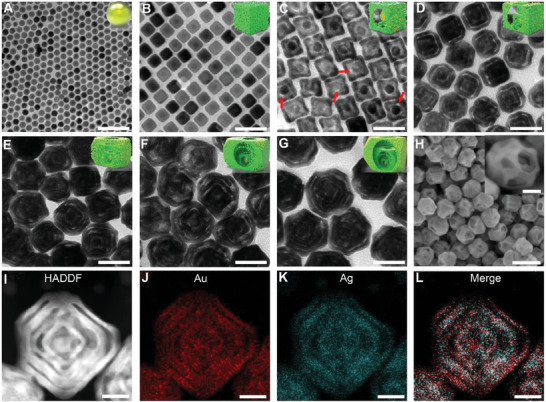
TEM images of CTAC‐capped Au seeds A), Au@Ag nanocubes with addition of 3 × 10^−4^ M AgNO_3_ B), resulting mono‐ C), bi‐ D), tri‐E), tetra‐F), and penta‐ G) Au@Ag‐Au NPs. The red arrow reprensted the asymmetric nanoholes. H) The SEM image, I) the HAADF‐STEM image, and J–L) elemental mapping of penta‐Au@Ag‐Au NPs. Scale bar: (A‐G) 50 nm, (H) 100 nm (inset 25 nm)), (I‐L) 20 nm.

**Figure 3:**
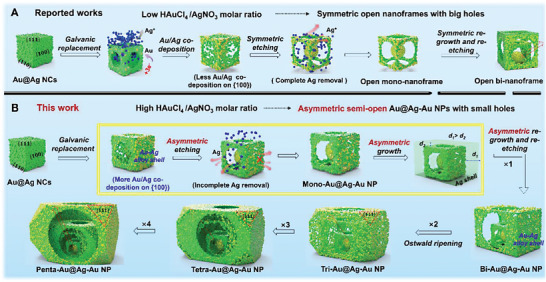
Schematic illustration of a proposed mechanism responsible for the transformation of traditional symmetric hollow nanoframes A) and asymmetric semi‐open multi‐Au@Ag‐Au NPs B).

Here, the number of galvanic replacement reaction and Ag growth, as well as the molar ratio of HAuCl_4_/AgNO_3_ were systematically controlled to obtain semi‐open multi‐Au@Ag‐Au core@carved‐shell superstructures (multi‐Au@Ag‐Au NPs), with multi‐layered built‐in nanogaps and asymmetric nanoholes. In detail, uniform Au@Ag nanocubes (Au@Ag NCs) were first prepared via the overgrowth of Ag on the as‐prepared cetyltrimethylammonium chloride (CTAC)‐capped Au seeds with an average diameter of 10 ± 0.6 nm (**Figure** **2**A,B and Figure S1, Supporting Information). The thickness of Ag shells allowed to systematically investigate the influence of shell thickness on the LSPR properties of the Au@Ag NCs. Figure 2B and Figure S2A–D, Supporting Information show TEM images of Au@Ag NCs collected from AgNO_3_ concentration increasing from 2 × 10^−4^ M to 10^−3^ M, where the edge length increased from 18 ± 2 nm to 31 ± 4 nm, and the LSPR peak redshifted from 410 nm to 430 nm (Figures S2I,K, Supporting Information). Second, the conformal overgrowth of Au shell on the Au@Ag NCs as a sacrificial template was implemented to produce carved core‐shell nanostructures in the presence of HAuCl_4_, CTAC, H_2_Asc, and NaOH at initial pH of 11.6. Typically, the Ag{100} facets selectively bonded by Cl^−^ could serve as an anode for the initiation of the galvanic replacement reaction, leading to the etching of Ag atoms from the side face.^[^ ^25^, ^26^ ^]^ The released Ag^+^ then remained soluble by complexing with Cl^−^ to form AgCl_2_^−^; meanwhile, sufficient Cl^−^ in the reaction medium could inhibit the formation of AuCl(OH)_3_^−^ and Au(OH)_4_^−^ ($AuCl_{4}^{-}+OH^{-}⇋AuCl{\left(OH\right)}_{3}^{-}+Au{\left(OH\right)}_{4}^{-}+Cl^{-}$) and thus keep the Au(III) precursor in the form of AuCl_4_^−^, maintaining a relatively high reduction potential.^[^ ^27^, ^28^ ^]^ Besides, H_2_Asc under alkaline conditions was neutralized into HAsc^−^, a much stronger reductant than H_2_Asc. Thus both AgCl_2_^−^ and AuCl_4_^−^ were quickly reduced to Ag ($AgCl_{2}^{-}\overset{HAsc^{-}}{\to }Ag+Cl^{-}$) and Au atoms ($AuCl_{4}^{-}+Cl^{-}+Ag\overset{HAsc^{-}}{\to }Au+AgCl_{2}^{-}$), leading to co‐deposition of them at the edges (Ag{110}) and corners (Ag{111}) of Au@Ag NCs.^[^ ^25^ ^]^ Finally, upon oxidative etching of residual Ag atoms with H_2_O_2_, the Au@Ag‐Au nanostructures with hollow nanoshells were formed. Figure S3A, Supporting Information shows the TEM image of resulting Au@Ag‐Au nanostructures synthesized with the molar ratio of HAuCl_4_/AgNO_3_ as 0.05. It was clear that symmetric hollow core‐shell nanoframes with open voids were produced. This is because at such a low molar ratio, the reduced Au and Ag atoms preferentially deposited onto the edges and corners of the original Au@Ag NCs, insufficient to generate Au‐Ag alloy,^[^ ^29^ ^]^ resulting big wall holes after Ag removal by H_2_O_2_ etching (**Figure** **3**A).

Generally, asymmetric nanostructures possess unique optical properties beyond those of symmetric ones.^[^ ^30^, ^31^, ^32^ ^]^ The giant localized field enhancement and high LSPR sensitivity of asymmetric plasmonic nanostructures have been demonstrated in previous work.^[^ ^33^, ^34^ ^]^ In order to obtain asymmetric multi‐layered nanostructures, we delivered a newly synthesis strategy by leveraging semi‐hollow Au@Ag NCs as a scaffold for further growth. Notably, tailoring the molar ratio of HAuCl_4_/AgNO_3_ is critical. Figure 2C and Figure S3B–D, Supporting Information show that the hole size of resulting Au@Ag‐Au nanostructures decreased with the molar ratio of HAuCl_4_/AgNO_3_increasing from 0.08 to 0.25. The reason is mainly that at a larger HAuCl_4_ amount, more reduced Au and Ag atoms can further diffuse onto the {100} facets, which would form a thick Au‐Ag alloy shell covering on the Au@Ag NCs surface;^[^ ^35^ ^]^ and the alloy shell could survive after H_2_O_2_ etching, leaving smaller nanoholes (Figure 3B). It should be noted that at the ratio of 0.16, semi‐open Au@Ag‐Au nanostructures with asymmetric nanoholes (labeled by red arrows in Figure 2C) were formed, which could be ascribed to asymmetric Ag etching due to ununiform surface coverage of Au‐Ag alloy.^[^ ^36^ ^]^ Accordingly, the LSPR peaks continuously blueshifted toward shorter wavelengths (Figure S3E, Supporting Information), while 520 nm assigned to the Au core appeared after etching of Ag and survived, which meant a coupling between Au NPs and Au‐Ag alloy shell would occur. Furthermore, using semi‐open mono‐Au@Ag‐Au NPs (Figure 2C and Figure S4, Supporting Information) as a scaffold, multiple processes including well‐faceted overgrowth of Ag, selective deposition of Au, and etching of residual Ag were systematically implemented (see Figure 3B). In Figure S5, Supporting Information, after Ag growth on mono‐Au@Ag‐Au NPs, the obtained nanostructures where the thickness of Ag shell was different can be clearly observed, which is fundamental to improve the asymmetry of superstructures.

Figure 2D–G shows the TEM images of bi‐, tri‐, tetra‐, and penta‐Au@Ag‐Au NPs obtained by respectively repeating the above process one to four times based on mono‐Au@Ag‐Au NPs. The resulting nanostructures which contained AuNP core at the center surrounded by two to five ultrathin metal layers respectively, maintained high uniformity in both morphology and size. The formation of truncated corner, i.e., new {111} facets were also observed in multi‐layered nanostructures (Figure 2G and 3B), which marked the start of dealloying,^[^ ^37^ ^]^ where the vacancies from the extraction of Ag atoms caused negative curvatures effect that was relieved by an internal Ostwald ripening process. Further statistical analysis revealed that the average size increased from 35 ± 4, 39 ± 4, 46 ± 7, 55 ± 8 to 72 ± 10 nm (Figure S6, Supporting Information). Moreover, corresponding UV‐vis spectra of each multi‐Au@Ag‐Au NPs were collected. Figure S6B shows after the formation of bi‐Au@Ag‐Au NPs, the LSPR peak blueshifted from 670 to 599 nm, while the peak at 520 nm still existed. This is because the contribution of residual Ag was greater than the size‐increase effect when the nanoparticle size was not large enough.^[^ ^38^ ^]^ The blueshifting effect dominated until the number of shells increased to three, while the LSPR peak exhibited only a single peak that shifted to 496 nm. With increase of the particle size, the effect of Ag become too diluted to overcome the redshifting effect resulting from larger NPs, resulting in broadband LSPR peaks of tetra‐Au@Ag‐Au NPs and penta‐Au@Ag‐Au NPs with the redshift to 589 nm and 650 nm respectively. Figure 2H displays irregular polygonal penta‐Au@Ag‐Au NPs with unique semi‐open structure, as demonstrated by the formation of asymmetric nanoholes on side faces (Figure 2H, inset). High‐angle annular dark‐field scanning TEM (HAADF‐STEM) and energy dispersive X‐ray spectroscopy mapping were further performed on an individual particle to examine the detailed structure, morphology and the spatial distributions of constituent elements. Multilayered built‐in nanogaps with 3 ± 2 nm in width were obviously observed in penta‐Au@Ag‐Au NPs consisting of Au‐Ag alloy that were not connected to each other. Moreover, both morphology and size of penta‐Au@Ag‐Au NPs remained uniform even if they were synthesized at three different batches (Figure S7, Supporting Information). Elemental mapping clearly showed the presence of Au and Ag atoms on the superstructures, while the Au atoms located dominantly at the center maintained the polygonal frame. The metal components of the multi‐core‐shell superstructures were thus Au‐enriched Au‐Ag alloy, as evidenced by the overlap of the elemental distributions.

### 2.2 Investigation of Plasmonic Properties of Multi‐Au@Ag‐Au NPs

**Figure 4:**
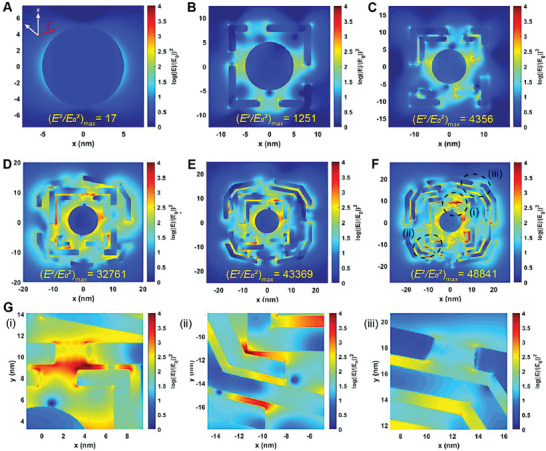
Electric‐field enhancement contour maps obtained from the FDTD calculations for the AuNPs A), mono‐ B), bi‐ C), tri‐ D), tetra‐ E), and penta‐ F) Au@Ag‐Au NPs. G) Partial enlarged details of hole‐to‐hole (i), shell‐to‐hole (ii), and shell‐to‐shell (iii) in penta‐Au@Ag‐Au NPs. The incident light was polarized along the *x*‐axis and propagated along the *z*‐axis.

To better understand the plasmonic properties of the multi‐Au@Ag‐Au NPs, finite‐difference time‐domain (FDTD) simulations was conducted to investigate distributions of the near‐field electromagnetic field (described by log(|*E*|/|*E*_0_|)^2^) at a fixed excitation wavelength range (400‐1000 nm). As shown in **Figure** **4**A,B, the maximum electromagnetic intensity ((*E* ^2^/*E*_0_^2^)_max_) of mono‐Au@Ag‐Au NP reached to 1251, ≈74 times higher than that of AuNP. With the number of shells increasing, strong hotspots and rich electromagnetic field distributions on multi‐Au@Ag‐Au NP occured, which showed potentially higher near‐field focusing ability (Figure 4A–E). Especially, the (*E* ^2^/*E*_0_^2^)_max_ value of penta‐Au@Ag‐Au NP was up to 48841 (Figure 4F), higher than that of most plasmonic nanostructures with interior nanogap reported by previous work (Table S1, Supporting Information). Besides, electromagnetic field distributions of penta‐Au@Ag‐Au NP with highly polarized electric surface charge density were intensively concentrated in rich plasmonic coupling sites involving shell‐to‐shell, shell‐to‐holes and hole‐to‐holes (Figure S8, Supporting Information; Figure 4G). In addition, when there was no nanoholes in penta‐Au@Ag‐Au NPs (Figure S9, Supporting Information), the strength of electromagnetic enhancement under the identical conditions decreased obviously ((*E* ^2^/*E*_0_^2^)_max_ = 36 100). This also emphasized the contribution of nanoholes to the intraparticle coupling enhancements. To experimentally compare the near‐field focusing capability of multi‐Au@Ag‐Au NPs, the SERS measurements using 4‐mercaptobenzoic acid (4‐MBA) as the model molecule were implemented. As can be seen in Figure S10A, Supporting Information, the signal intensity at peak 1590 cm^−1^ increased dramatically with increasing the number of shells, which can be attributed to enhanced inner coupling effect, as evidenced by FDTD simulation results. Among them, penta‐Au@Ag‐Au NPs showed much‐enhanced signal with the peak intensity reaching ∼5.85, ∼3.68, ∼1.45 and ∼1.13 fold of those from four other core‐shell Au@Ag‐Au NPs respectively (Figure S10B, Supporting Information), which emphasized the critical role of the nanoholes, multiple shells and nanogaps in affording optimal SERS activities. The calculated electromagnetic enhancement factor of Raman signals obtained from penta‐Au@Ag‐Au NPs was calculated to be 3.23 × 10^9^ (Figure S11, Supporting Information), indicating an excellent SERS enhancing performance.

### 2.3 Investigation of Optical Adaptation to Different Raman Lasers

**Figure 5:**
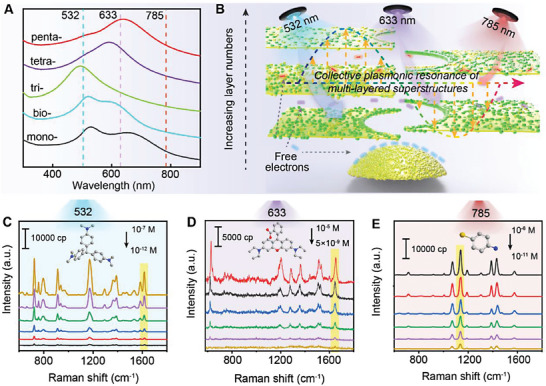
A) The schematic diagram of light‐confinement effect due to collective plasmonic resonance of multi‐layered superstructures. B) Increasing layer numbers caused wavelength overlaps between the LSPR absorption and Raman lasers. SERS spectra of C) CV with concentrations ranging from 10^−12^ M to 10^−7^ M at 532 nm excitation source, D) rhodamine B with concentrations ranging from 5 × 10^−9^ M to 10^−5^ M at 633 nm excitation source, and E) 4‐aminothiophenol with concentrations ranging from 10^−11^ M to 10^−6^ M at 785 nm excitation source.

Further simulated extinction results revealed that multi‐Au@Ag‐Au NPs displayed layer‐dependent plasmonic properties in terms of plasmon resonance wavelengths (Figure S12, Supporting Information), which were generally in good agreement with the experimental ones. According to simulation results, penta‐Au@Ag‐Au NPs could support strong electromagnetic resonances over a wide wavelength region. This was relevant to a broad LSPR absorption spectrum of the superstructure nanoparticles due to the joint contribution of multiple layers, which may result in large wavelength overlap between absorption band and typical used Raman lasers (**Figure** **5**A,B). Therefore, to investigate the light‐harvesting capability under different lasers, SERS spectra of crystal violet (CV), rhodamine B, and 4‐aminothiophenol molecules on substrates were collected at 532, 633, and 785 nm respectively, as shown in Figure 5C,D. The SERS spectral profiles of three molecules with different concentrations were clearly detected under the representative larser. In detail, crystal violet (10^−12^∼10^−7^ M) showed characteristic peak at 1625 cm^−1^ assigned to aromatic C−C stretching;^[^ ^39^ ^]^ rhodamine B (5 × 10^−9^∼10^−5^ M) exhibited distinct peak at 1650 cm^−1^ referred to C═C stretching,^[^ ^40^ ^]^ and 4‐aminothiophenol (10^−11^∼10^−6^ M) had the peak at 1078 cm^−1^ attributed to C−S and C−C ring stretching.^[^ ^41^ ^]^ Therefore, adopting penta‐Au@Ag‐Au NPs as the SERS substrates would be promising for achieving broadband and sensitive detection, which is mainly due to the favorable light‐harvesting capability of penta‐Au@Ag‐Au NPs that were adaptive to wide excitation sources.

**Figure 6:**
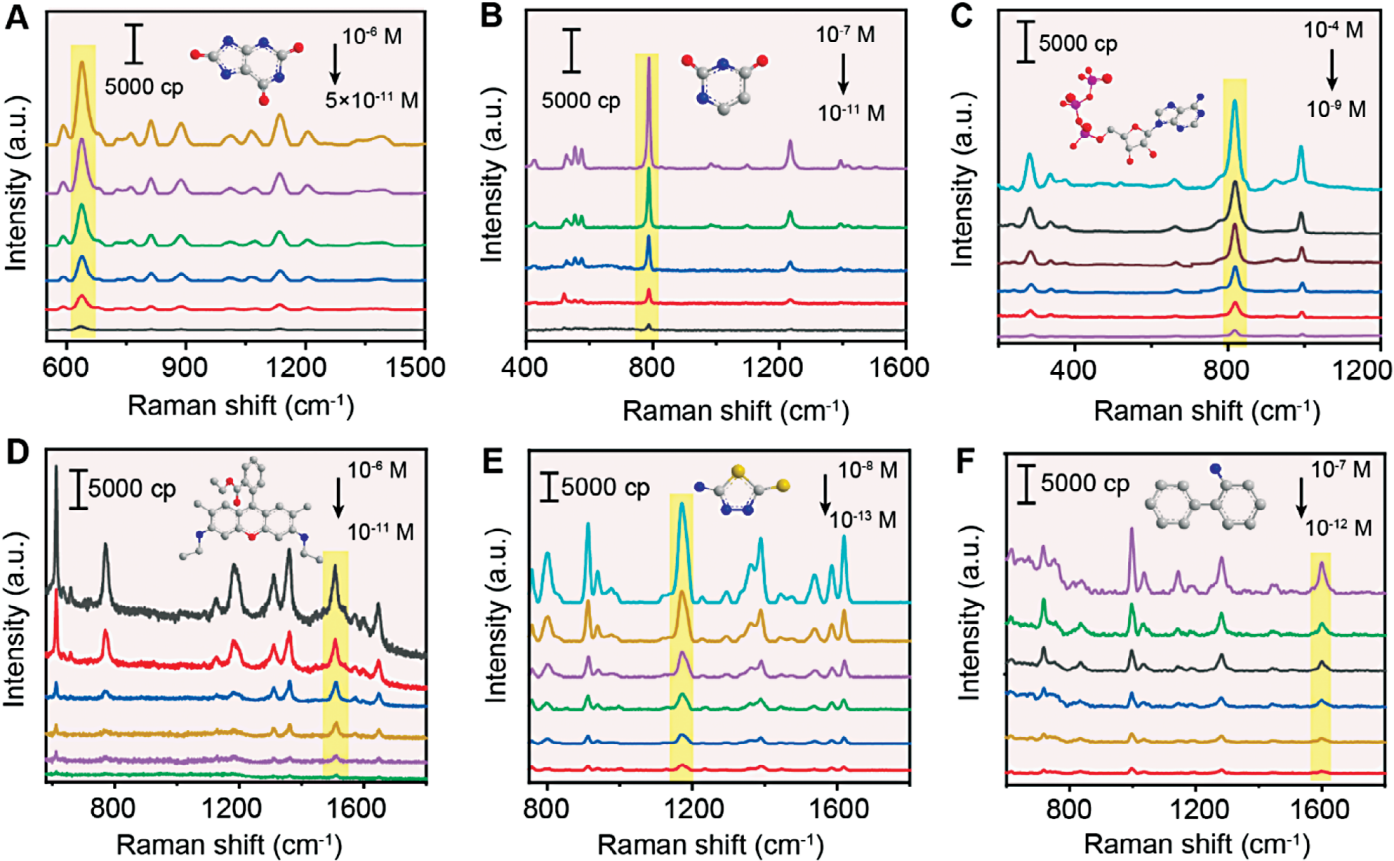
The investigation of SERS performance of penta‐Au@Ag‐Au NPs using 785 nm laser. SERS spectra of typical biological molecules: A) uric acid with concentrations ranging from 5 × 10^−11^ M to 10^−6^ M, B) uracil with concentrations ranging from 10^−11^ M to 10^−7^ M, C) adenosine triphosphate with concentrations ranging from 10^−9^ M to 10^−4^ M, D) R6G with concentrations ranging from 10^−11^ M to 10^−6^ M, E) thiediazole copper with concentrations ranging from 10^−13^ M to 10^−8^ M, and F) 2‐aminobiphenyl with concentrations ranging from 10^−12^ M to 10^−7^ M. The characteristic peaks of molecules were labeled by yellow rectangle.

Furthermore, using 785 nm (one of the most common wavelengths) as the irradiation laser,^[^ ^42^ ^]^ penta‐Au@Ag‐Au NPs were adopted to evaluate the practicability of detecting various molecules including biological metabolites (uric acid, uracil, adenosine triphosphate),^[^ ^43^, ^44^, ^45^ ^]^ dyes (rhodamine 6G, R6G),^[^ ^46^ ^]^ pesticide residues (thiediazole copper),^[^ ^47^ ^]^ and organic precursors (2‐aminobiphenyl).^[^ ^48^ ^]^ As shown in **Figure** **6**, the spectra could be detected even when the concentration of uric acid, uracil, adenosine triphosphate, R6G, thiediazole copper, and 2‐aminobiphenyl decreased to 5 × 10^−10^ M, 10^−11^ M, 10^−9^ M, 10^−11^ M, 10^−13^ M, and 10^−12^ M, respectively. In Figure S13, Supporting Information, the plot (in log scale) for variation in Raman peak intensity with molecules concentrations showed clear linear trends. The vibrational bands of the different molecules were prominently marked in figures and were assigned according to previously reported works (Table S2, Supporting Information). It was also observed that highly sensitive detection could be achieved regardless of molecules with a weak affinity for the substrate, such as uracil and adenosine triphosphate, revealing a versatility of plasmonic penta‐Au@Ag‐Au NPs for real‐world applications.

### 2.4 Analysis of the Feasibility of Molecules Captured into Hotspots Center

**Figure 7:**
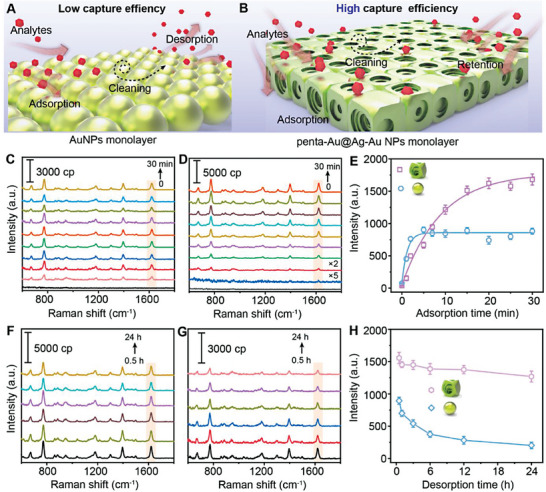
The schematic diagram of assembled AuNPs A) and penta‐Au@Ag‐Au NPs B) monolayer is used for dynamic monitoring of the adsorption and desorption of MB. Compared with AuNPs, penta‐Au@Ag‐Au NPs due to the specific structure could capture MB molecules and retain them in interior nanogaps. Time evolution of SERS spectra after MB adsorption by AuNPs C) and penta‐Au@Ag‐Au NPs D) monolayer. E) Growth of MB peak (1621 cm^−1^) versus time. Time evolution of SERS spectra after MB desorption from penta‐Au@Ag‐Au NPs F) and AuNPs G) monolayer. H) Decline of MB peak (1621 cm^−1^) versus time. The SERS spectra are representative results from three independent experiments.

In addition to the light‐harvesting ability and the high electromagnetic field strength of penta‐Au@Ag‐Au NPs, the specific semi‐open and multi‐layered structure motivated us to explore the potential of effectively capturing and activating analytes assisted by the instant interfacial assembly technology.^[^ ^49^ ^]^ Taking water‐soluble methylene blue (MB) as the adsorption model molecule, the capture effiencies of penta‐Au@Ag‐Au NPs and AuNPs were evaluated and compared (Figure S14, Supporting Information). As shown in **Figure** **7**A,B, and Figure S15, Supporting Information, after hexane evaporation, self‐assembled NPs monolayers at the interface were formed, leaving a light bule water phase containing residual MB. It can be seen that the color resulted from penta‐Au@Ag‐Au NPs seemed lighter, as compared with that of AuNPs (Figure S16, Supporting Information). Meanwhile, the corresponding UV‐vis absorption spectra revealed that the capture efficiency of penta‐Au@Ag‐Au NPs (41.5%) was much better than that of Au NPs (20.7%) (Figure S15C, Supporting Information). Moreover, NPs monolayers were used to provide Raman fingerprint information of the adsorption and desorption kinetics of MB. As shown in Figure 7C,D, Supporting Information, when monolayers were immersed in MB/water solution, initially no band was observed but the characteristic peak at 1621 cm^−1^ gradually growed over time and reached a plateau within 5 min using AuNPs. In contrast, the time of adsorption equilibrium was extended to 15 min using penta‐Au@Ag‐Au NPs, while the peak intensity was twice higher than that of AuNPs (Figure 7E). Further desorption experiments showed that after soaking cleaning overnight, the signal intensity of MB fluctuated slightly using penta‐Au@Ag‐Au NPs, whereas a 75% decrease was observed on AuNPs (Figure 7F–H). These results indicated that penta‐Au@Ag‐Au NPs owing to the presence of abundant built‐in nanogaps and wall holes effectively allowed the effect of non‐equilibrium binding to be minimized and further enabled sufficient adsorption of MB into hotspots center where electromagnetic near‐field was largely enhanced.

### 2.5 Investigation of the Possibility of Effective Activiation of Molecules

**Figure 8:**
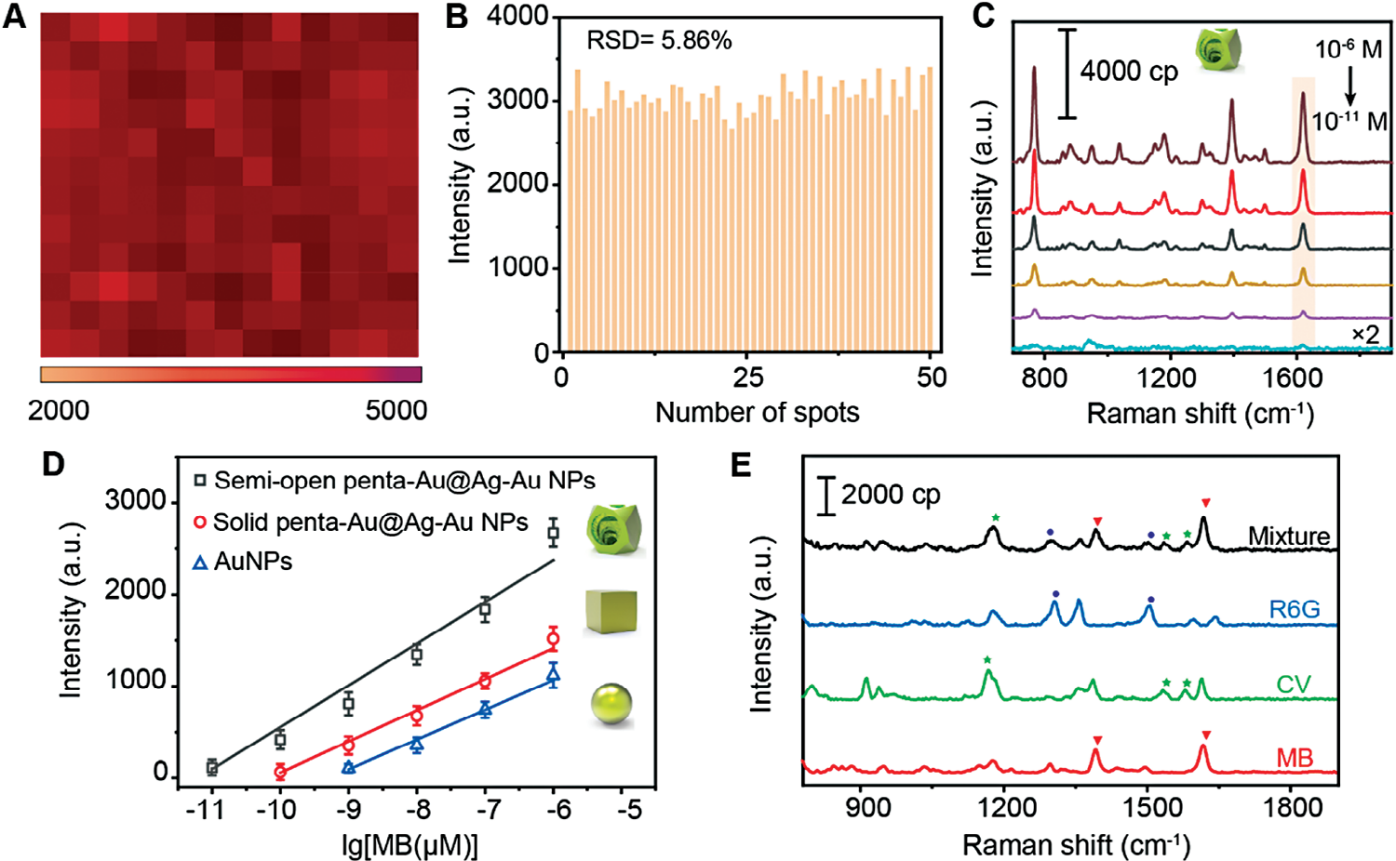
A) The SERS imaging of assembled penta‐Au@Ag‐Au NPs monolayer with addition of 10^−7^ M MB. B) SERS intensity of the peak at 1621 cm^−1^ collected from different spots on penta‐Au@Ag‐Au NPs monolayer. C) SERS spectra of MB with different concentrations using semi‐open penta‐Au@Ag‐Au NPs monolayer. D) SERS intensity of the peak at 1621 cm^−1^ as a function of the logarithm concentrations of MB collected from semi‐open penta‐Au@Ag‐Au NPs, solid penta‐Au@Ag‐Au NPs, and AuNPs. E) SERS spectra of various molecules, namely, 10^−7^ M MB (red), 10^−7^ M CV (green), 10^−7^ M R6G (blue), and their mixture (black). The concentration of each molecule was 10^−7^ M MB, 10^−8^ M CV, and 10^−8^ M R6G in the mixture. The symbols mark the characteristic bands contributed by MB (triangle), CV (star), and R6G (circle). The SERS spectra are representative results from three independent experiments.

In SERS imaging results of penta‐Au@Ag‐Au NPs monolayer where MB was captured (**Figure** **8**A), the uniform mapping color reflected a small fluctuation of SERS intensity with the relative standard deviation as low as 5.86% (Figure 8B). Encouraged by the high reproducibility and favorable capture efficiency, the capability of SERS activiation using penta‐Au@Ag‐Au NPs was investigated. As shown in Figure 8C, the SERS signal of MB increased as a function of concentration from 10^−6^ to 10^−11^ M; thus, a linear relationship between the characteristic peak at 1621 cm^−1^ and lg C_MB_ was obtained (Figure 8D). The estimated limit of detection (LOD)LOD was 3.22 × 10^−12^ M (S/N = 3). Meanwhile, to well understand activation behavior of penta‐Au@Ag‐Au NPs, solid penta‐Au@Ag‐Au NPs (interior nanogaps filled with Ag) and AuNPs monolayers were investigated under identical conditions. As shown in Figure 8D and Figure S17, Supporting Information, LODs obtained from solid penta‐Au@Ag‐Au NPs and AuNPs were 5.35 × 10^−11^ M and 8.26 × 10^−9^ M respectively. Because solid NPs without wall holes and intra‐nanogaps was incapable of trapping molecules effectively, the signal was highly dependent on interparticle coupling. In addition to interparticle plasmon field coupling, penta‐Au@Ag‐Au NPs were capable of hosting collective plasmon oscillations distributed among nanoholes and different metallic layers as well as built‐in nanogaps, which demonstrated a considerable SERS activation of molecules. Moreover, we investigated the feasibility of detecting multiple chemical analytes. Figure 8E shows that the SERS spectra of other two representative dyes (CV and R6G) were readily obtained. Their unique SERS bands matched well with the results in Figure 6A, and were obviously distinguished in a mixture. All demonstrations confirmed that penta‐Au@Ag‐Au NPs were not only responsible for affording high‐density hotspot for SERS analysis, but for effectively capturing and activating molecules of interest in a trace amount.

## 3 Conclusion

In summary, we offered a newly pathway to design semi‐open multi‐Au@Ag‐Au NPs with controllable layer numbers and asymmetric nanoholes, which achieved strong electromagnetic near‐field enhancement and effective SERS activation of analytes. Due to multimodal plasmonic coupling effects from carved nanoholes, intra‐nanogaps between adjacent metallic layers, the electromagnetic field strength and enhancement factor of the penta‐Au@Ag‐Au NPs up to 48 841 and 3.23 × 10^9^ could be obtained. Furthermore, penta‐Au@Ag‐Au NPs showed a desirable ability of light harvesting over wide excitation sources, which realized highly quantitative and sensitive detection of various molecules with representative excitation wavelength. Owing to the presence of outer carved nanoholes of penta‐Au@Ag‐Au NPs, target molecules without functional groups could be effectively delivered and captured into multilayered interior gaps. Moreover, the assembled penta‐Au@Ag‐Au NPs monolayer with high signal reproducibility demonstrated extraordinary SERS activation of analytes due to the combination of inter‐ and intra‐particle electromagnetic coupling. Taken together, this work provides a new way for designing plasmonic nanostructures to enhance light‐matter interaction and near‐field focusing, as well as to actively capture target molecules into optimal hotspots, thereby expanding the applications in environmental, biological, and chemical fields where the ultrasensitive detection is urgently needed.

## 4 Experimental Section

### Materials

Chloroauric acid (HAuCl_4_·3H_2_O, ≥ 99.9%), rhodamine B (≥ 99.9%), rhodamine 6G (≥ 99.9%), and L‐ascorbic acid (H_2_Asc, ≥ 99.5%) were bought from Sigma–Aldrich (USA). Cetyltrimethylammonium bromide (CTAB, ≥ 99.0%) and cetyltrimethylammonium chloride (CTAC, ≥ 99.0%) were procured from TCI (Shanghai, China). 4‐Aminothiophenol (≥ 97.0%) was obtained from Alfa Aesar (Shanghai, China). 5‐Amino‐2‐thiol‐1,3,4‐thiadiazole (≥ 98%), 2‐aminobiphenyl (≥ 95%), crystal violet (≥ 90%), and methylene blue (≥ 90%) were bought from Aladdin (Shanghai, China). Silver nitrate (AgNO_3_, > 99.0%), absolute ethanol, and hydrogen peroxide (H_2_O_2_, 30.0%) were procured from Sinopharm Chemical Reagent Co., Ltd. (Shanghai, China). All reagents were used without further purification. Deionized (DI) water (resistivity 18.25 MΩ cm at 25 °C) was used for all experiments. All glassware were soaked in fresh Aqua Regia (v/v (HCl/HNO_3_) = 3/1) for 30 min, rinsed with a plenty of DI water, and dried at 60 °C for further use.

### Apparatus

UV−vis spectra of samples in aqueous solutions were measured on a TU‐1810 spectrophotometer (Purkinje General Instrument Co, China). HT‐7700 (Hitachi, Japan) and Talos F200X instruments (Thermo Fisher Scientific, USA) operated at an acceleration voltage of 200 kV were used to acquire transmission electron microscopy (TEM) images and high‐angle annular dark‐field scanning transmission electron microscopy (HAADF‐STEM) images. Scanning electron microscopy (SEM) images were taken using a Supra55 field emission microscope operated at an acceleration voltage of 15 kV (Zeiss, Germany). SERS signals were recorded on a LabRAM Odyssey Raman spectrometer (Horiba, Japan) coupled to 785 and 633 nm lasers.

### Synthesis of Au@Ag Nanocubes (Au@Ag NCs)

For the preparation of Au@Ag NCs, the seed‐mediated growth method reported by Xia's group was employed with a slight modification.^[^ ^25^ ^]^ Simply, 3 mL of CTAC‐stabilized AuNPs, 3 mL of 200 mM CTAC aqueous solution and series of volumes of 100 mM AA aqueous solution were mixed in a 20 mL vial. After the mixture was mixed at 65 °C for 10 min under magnetic stirring, slowly add a series of volumes of 10 mM AgNO_3_. Afterward, the aqueous solution turned from peach red to yellow. Then the obtained solution was kept in a water bath at 65°C for 4 h and naturally cooled down to room temperature. Finally, the obtained solution was centrifuged at 10 000 rpm, washed twice with ultrapure water, and dispersed in an equal volume of ultrapure water.

### Synthesis of Multi‐Au@Ag‐Au Nanoparticles (Multi‐Au@Ag‐Au NPs)

For the galvanic replacement process, 50 µL of 10 mM NaOH aqueous solution, 50 µL of 100 mM fresh H_2_Asc aqueous solution, and 50 µL of 10 mM HAuCl_4_ aqueous solution were quickly injected into a mixture consisting 1 mL of 100 mM CTAC solution and 1 mL as‐prepared Au@Ag NCs while vigorously stirring. After the mixture was heated at 30 °C for 30 min, the unreacted reagents were then removed by using centrifugation (12 000 rpm for 10 min). Followed by addition of 30 µL of H_2_O_2_ (30% v/v) with stirring for 12 h at room temperature, the resulting mono‐Au@Ag‐Au NPs were obtained by centrifugation at 12 000 rpm for 15 min. The above Ag growth and galvanic replacement were repeated different times to synthesize multi‐Au@Ag‐Au nano‐superstructure.

### Finite‐Difference Time‐Domain Simulations

During the simulation, an excitation light ranging from 400 to 1000 nm was launched into the region of the nanoparticles, with the direction set to be perpendicular to the substrates. A mesh size of 1 nm was employed for acquiring the extinction spectra and electric field contours of multi‐Au@Ag‐Au NPs. The incident light was polarized along the x‐axis and propagated along the z‐axis. Perfectly matched layer (PML) boundary condition was used to simulate the infinite space surrounding the nanostructures. The PML was placed far enough to fit the largest value of optical extinction cross section. For simplification, AuNP was constructed as a model, which was surrounded by water with a refractive index of 1.33. Successively, AuNP surrounded by multiple shell with irregular nanoholes were constructed based on real morphologies depicted in TEM and SEM results. The dielectric function of Au was taken from Johnson and Christy's data. For simplification, the nanostructures were assumed to be made of pure Au, which has been widely adopted for the numerical simulation of plasmonic properties of AuAg alloyed nanostructures.^[^ ^28^, ^36^ ^]^

### Instant Interfacial Assembly Technology for Penta‐Au@Ag‐Au NPs Assembly

1 mL newly penta‐Au@Ag‐Au NPs and 4 mL DI water were injected into a 20‐mL glass bottle. Then 5 mL ethanol/hexane (v/v = 2:1) containing PFT (1 mM) was quickly poured into the above NPs solution. Followed by immediately oscillating for 5 s, the mixture was left to form dense 2D NPs monolayer. After overnight of hexane evaporating, the monolayer was transferred to silicon plates for further use. Other multi‐ Au@Ag‐Au NPs and AuNPs monolayers were accquired using the same procedure.

### Evaluation of the Capture Efficiency

MB with a certain amount was rapidly injected into water phase of the above NPs monolayer (with hexane left), immediately followed by a continuous oscillation. MB were captured into inter‐ or intra‐nanogaps during the oscillating process. After overnight of hexane evaporating, the UV‐vis absorption spectra of water phase were collected to evaluate and compare the capture efficiency of penta‐Au@Ag‐Au NPs and Au NPs.

### The Investigation of Adsorption and Desorption of MB

The as‐prepared penta‐Au@Ag‐Au NPs and AuNPs monolayer was cleaned by O_2_ plasma with a plasma cleaner for 5 min at 60 W to remove the adsorbed organic molecules. Then, the silicon plates covered by monolayers were immersed into MB (10^−7^ M) solution loaded in a container. Following that, Raman spectra of the same point on the monolayers were collected at a certain interval. After that, the desorption of MB was implemented by continuously immersion cleaning in ethanol. Raman spectra were also collected according to the mentioned above.

### SERS Measurements

The as‐prepared NPs (including multi‐Au@Ag‐Au NPs, solid penta‐Au@Ag‐Au NPs, and AuNPs) monolayers were cleaned by O_2_ plasma with a plasma cleaner for 5 min at 60 W to remove the adsorbed organic molecules. Then, the substrates were put into a glass container in which a certain concentration of molecules was subsequently added. Following that, the whole container was then kept at room temperature for 30 min for sufficient adsorption. The substrates were then rinsed with ethanol and water three times respectively to remove molecules with non‐equilibrium adsorption. SERS spectra were collected by a 50× microscope objective with 0.5 NA. The acquisition time of each spectrum was 5 s with two accumulations. Laser powers at the sample of 20 mW at 532 nm, 4 mW at 633 nm and 20 mW at 785 nm were measured.

## Conflict of Interest

The authors declare no conflict of interest.

## Supporting information

Supporting Information

## Data Availability

The data that support the findings of this study are available from the corresponding author upon reasonable request.
