# Supplementary material for: Plasmonic Multi‐Layered Built‐in Hotspots Nanogaps for Effectively Activating Analytes
Source: Adv Sci (Weinh). 2023 Dec 3;11(7):2306125. doi: 10.1002/advs.202306125 (PMC10870027; doi:10.1002/advs.202306125)
Supplement: Supplementary file 1 — Supporting Information [file ADVS-11-2306125-s001.pdf]

ADVANCED SCIENCE
Open Access

Supporting Information

for *Adv. Sci.*, DOI 10.1002/advs.202306125

Plasmonic Multi-Layered Built-in Hotspots Nanogaps for Effectively Activating Analytes

Lei Jiang, Xiaoyuan Wang, Jingyi Zhou, Qianqian Fu, Bihu Lv, Yixuan Sun, Liping Song and Youju Huang**

Supporting Information

Plasmonic Multi-Layered Built-in Hotspots Nanogaps for Effectively Activating Analytes

Lei Jiang[#], Xiaoyuan Wang[#], Jingyi Zhou, Qianqian Fu, Bihu Lv, Yixuan Sun, Liping Song, and Youju Huang**

S1. Synthesis of Au seeds

The Au spherical seeds were prepared in two steps. (1) Synthesis of CTAB-stabilized Au nanocrystalline: 0.6 mL of 10 mM ice $NaBH_4$ aqueous solution was quickly injected into a mixture consisting of 9.9 mL of 100 mM CTAB solution and 100 μL of 10 mM $HAuCl_4$ under rapid stirring for 3 min. The obtained solution was then kept in a water bath at 30 °C for 3 h for maturation before use. (2) Synthesis of CTAC-stabilized Au seeds: 50 μL of CTAB-capped Au nanocrystalline was mixed with 1.5 mL of 100 mM ascorbic acid and 2.0 mL of 200 mM CTAC under gentle stirring, followed by immediate addition of 2.0 mL of 0.5 mM $HAuCl_4$. After gentle stirring for 15 min, the obtained Au seeds were purified by centrifugation and the pellet was redispersed in DI water.

S2. The equations involved in the galvanic replacement and co-deposition process

$$\mathrm{CTAC} \rightarrow \mathrm{CTA^{+}} + \mathrm{Cl^{-}} \quad (1)$$

$$\mathbf{AuCl_4^-} + \mathrm{OH^-} \leftrightharpoons \mathrm{AuCl(OH)_3^-} + \mathrm{Au(OH)_4^-} + \mathbf{Cl^-} \quad (2)$$

$$\mathrm{H_2Asc} + \mathrm{OH^-} \rightarrow \mathbf{HAsc^-} + \mathrm{H_2O} \quad (3)$$

$$\mathrm{AuCl_4^-} + \mathrm{Cl^-} + \mathrm{Ag} \xrightarrow{\mathrm{HAsc^-}} \mathbf{Au} + \mathrm{AgCl_2^-} \quad (4)$$

$$\mathrm{AgCl_2^-} \xrightarrow{\mathrm{HAsc^-}} \mathbf{Ag} + \mathrm{Cl^-} \quad (5)$$

S3. Calculation of Enhancement Factor

The enhancement factor of Raman signals was based on the following equation:

$$EF = \frac{I_{SERS}}{I_{Raman}} \cdot \frac{N_{Raman}}{N_{SERS}}$$

where I_{SERS} and I_{Bulk} represent the intensities of 4-MBA in SERS (1583 cm^{-1}) and normal Raman spectra (1595 cm^{-1}), the intensity ratios of I_{SERS}/I_{Raman} are about 5.17 according to Figure S9, where N_{Raman} and N_{SERS} represent the number molecules in the bulk sample and the monolayer of penta-Au@Ag-Au NPs closely packed on the sample surface.

$$N_{Raman} = \frac{\pi r^2 \cdot h \cdot \rho}{M_W} N_A$$

where ρ is the density of bulk 4-MBA (1.26 g/cm^3); Mw is the molecular weight of 4-MBA (152.17 g/mol); NA is Avogadro's constant (6.02 × 10^{23} mol^{-1}). We used a 785 nm laser

(21 mW) focused through an objective lens (50×, NA=0.5). Assuming the excitation volume as a cylinder, the radius of the laser beam (r) was 2 μm (instrument parameters), and the laser focal depth (h) was calculated by:

$$\frac{h}{2r} = \frac{3.28\eta}{N.A.}$$

where η is the refractive index of the silicon wafer (3.42) and r is the radius of the laser beam (~2 μm). The laser focal depth *h* is calculated as ~45 μm. Therefore, the calculated number of N_{Raman} is 5.42×10^{11}.

N_{SERS} was calculated by the following equation:

$$N_{SERS} = A \cdot D \cdot N_A$$

where A is the SERS active area on one penta-Au@AuAg NP that can be adsorbed by 4-MBA (estimated as 2.83×10^{-15} m^2), and D is the coverage density of 4-MBA molecules on penta-Au@Ag-Au NPs (estimated as 5.10×10^{-7} mol/m^{-2}). Therefore, the calculated number of N_{Raman}/N_{SERS} is 6.24×10^8; the EF of penta-Au@Ag-Au NPs is calculated to be $\mathbf{3.23 \times 10^9}$, indicating that the proposed scale-up multi-Au@AuAg NPs have excellent SERS enhancing performance.

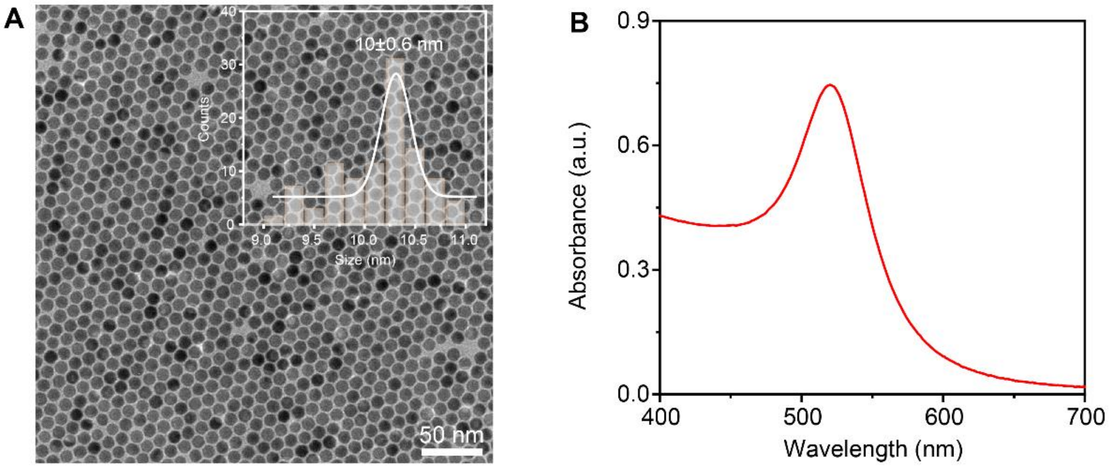

Figure S1 The TEM image (A) and UV-vis spectra (B) of CTAC-capped Au seeds. Inset in A: Statistical size distribution of Au seeds.

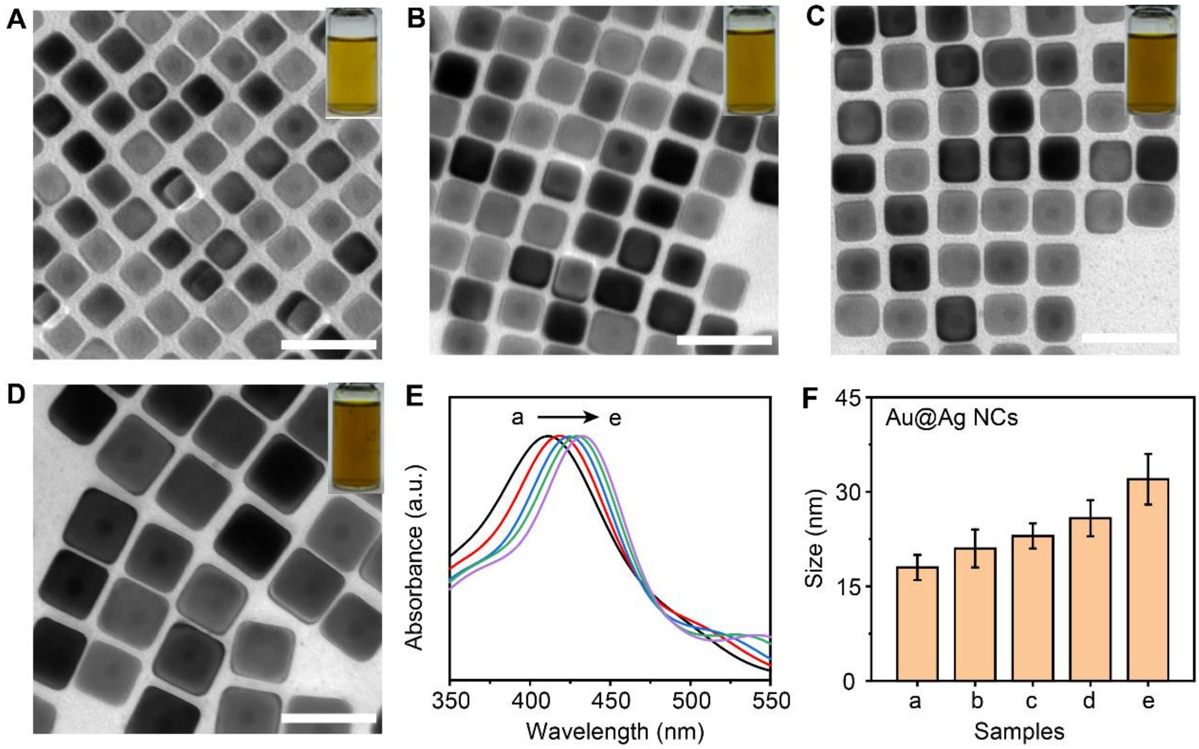

Figure S2 TEM images of Au@Ag NCs (A-D). The Au@Ag NCs samples were prepared using the standard protocol by titrating different concentrations of $AgNO_3$ into an aqueous suspension of Au seeds in the presence of CTAC and H_2Asc: 2×10^{-4} M (A), 4×10^{-4} M (B), 6×10^{-4} M (C), and 10^{-3} M (D), respectively. Inset: optical photographs. Corresponding UV-vis spectrum (E) and statistical size distribution of Au@Ag NCs (F) (a-e: 2×10^{-4} M, 4×10^{-4} M, 6×10^{-4} M, 8×10^{-4} M and 10^{-3} M). Scale bar: 50 nm.

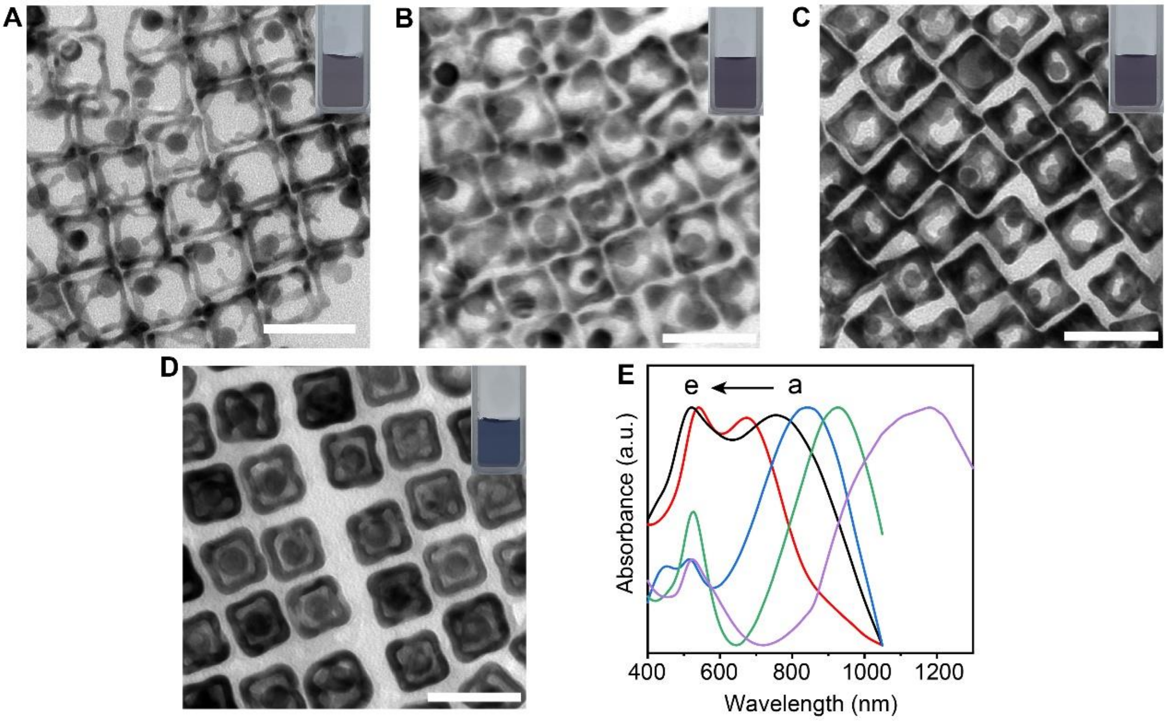

Figure S3 TEM images of mono-Au@AuAg samples (E-H). Samples were prepared by titrating $HAuCl_4$, H_2Asc, NaOH, and CTAC into aqueous suspension of Au@Ag NCs upon different molar ratio of $HAuCl_4/AgNO_3$: 0.05 (A), 0.08 (B), 0.125 (C), and 0.25 (D). Inset: optical photographs. Corresponding UV-*vis* spectrum of mono-Au @AuAg (E) (a-e: 0.05, 0.08, 0.125, 0.16, 0.25). Scale bar: 50 nm.

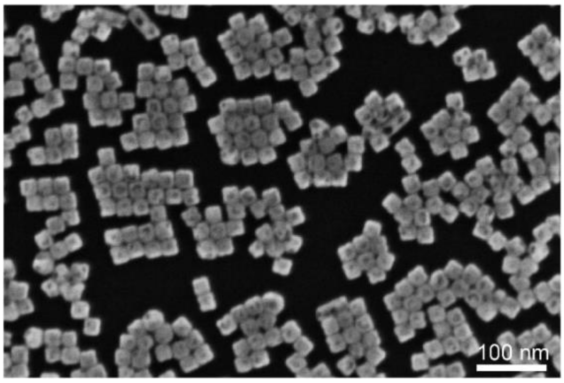

Figure S4 The SEM image of mono-Au@AuAg NPs produced by Au@Ag NCs with the molar ratio of 0.16.

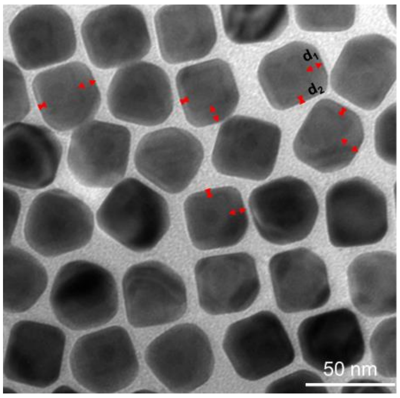

Figure S5 The SEM images of mono-Au@AuAg NPs after the addition of $AgNO_3$.

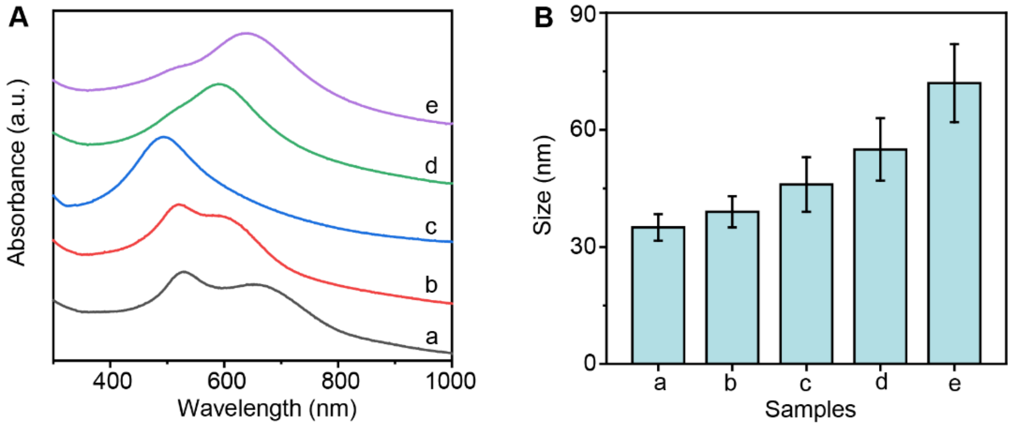

Figure S6 UV-vis spectra (A) and statistical size distribution (B) of mono- (a), bi- (b), tri- (c), tetra- (d), and penta- (e) Au@Ag-Au NPs.

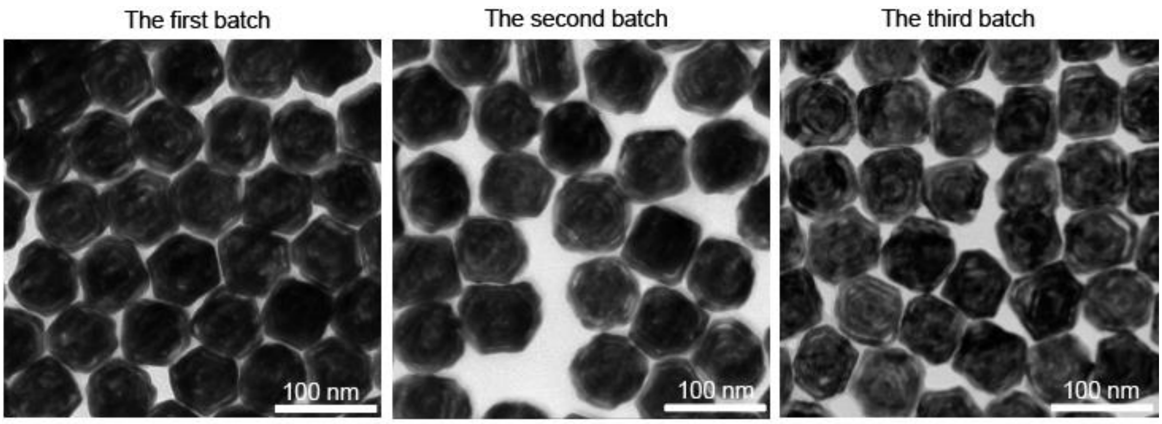

Figure S7 The TEM images of three different batches of penta-Au@Ag-Au NPs.

Table S1 The comparison of electromagnetic field intensity between the penta- Au@AuAg NPs and other reported nanostructures with interior nanogap.

$(E^2/E_0^2)_{max}$	Simulation method	Boundary conditions	Wavelengths	Polarization direction	References

**48841**	**FDTD**	**Perfectly matched layer (PML) and scattering**	**400-1000 nm**	***x* axis**	**This work**
10787	FDTD	Not mentioned	818 nm	Not mentioned	*Nano Lett.* 2016, 16, 3675−3681
316	FDTD	Not mentioned	400-1200 nm	Not mentioned	*Small* 2019, 15, 1902608
87000	FDTD	PML	633 nm	Not mentioned	*Angew. Chem.* 2019, 131, 16037–16041
1000	FDTD	Not mentioned	400-1000 nm	*x* axis	*Nanoscale Horiz.*, 2022, 7, 554–561
1000	FDTD	Not mentioned	785 nm	*x* axis	*ACS Sens.* 2022, 7, 3126−3134
625	FEM	Not mentioned	785 nm	*y* axis	*J. Am. Chem. Soc.* 2021, 143, 15113−15119
900	FEM	PML	1138 nm	*x* axis	*Adv. Funct. Mater.* 2021, 2103186
10000	FEM	Not mentioned	300-2000 nm	*x* axis	*Nat. Commun.* 2022, 13, 5249

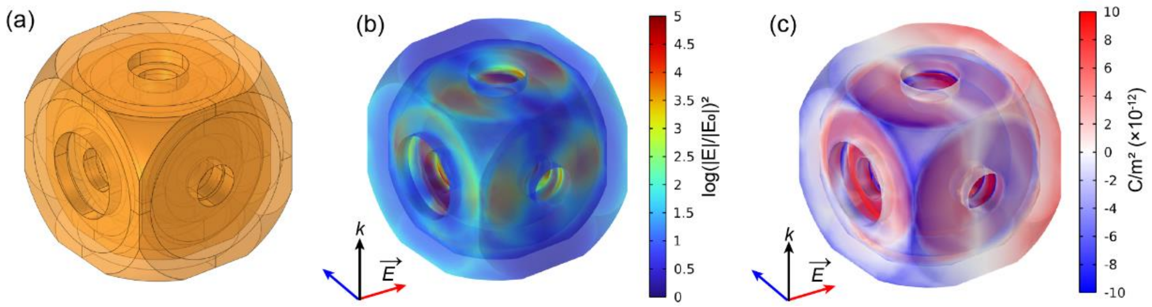

Figure S8 The 3D model (a), the simulation of 3D electromagnetic field distribution (b) and surface charge density (c) for penta-Au@Ag-Au NPs.

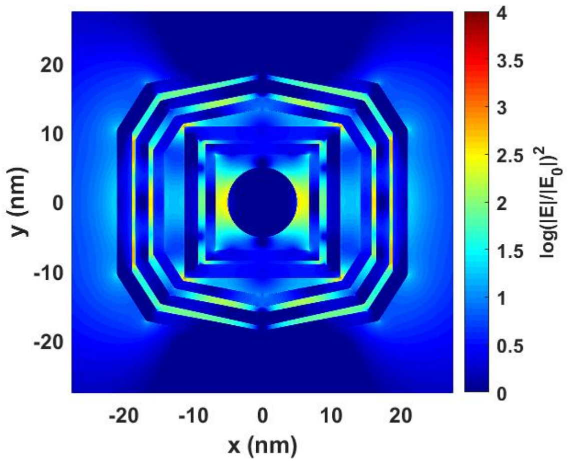

Figure S9 Electric-field enhancement contour map obtained from the FDTD calculations for penta-Au@Ag-Au NPs without nanoholes.

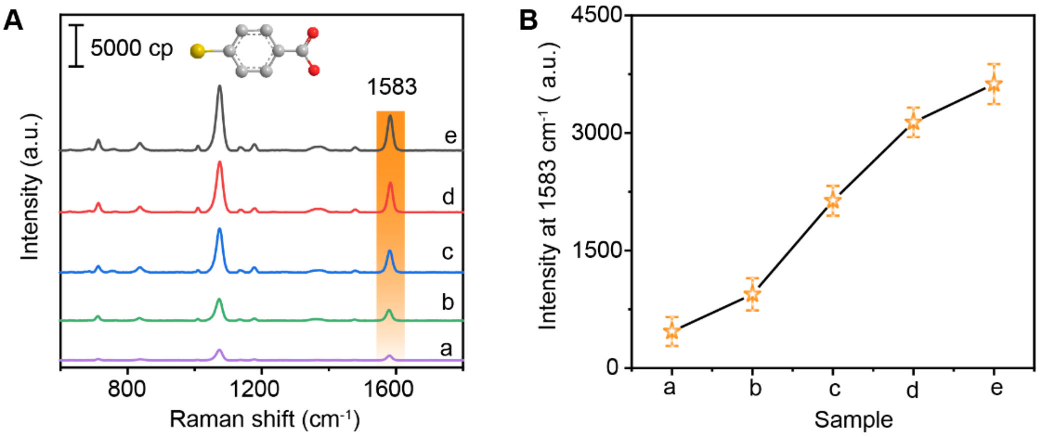

Figure S10 (A) SERS spectra of 4-MBA collected from mono- (a), bi- (b), tri- (c), tetra- (d), and penta- (e) Au@Ag-Au NPs. (B) Intensity of peak at 1583 cm^{-1} corresponding to A.

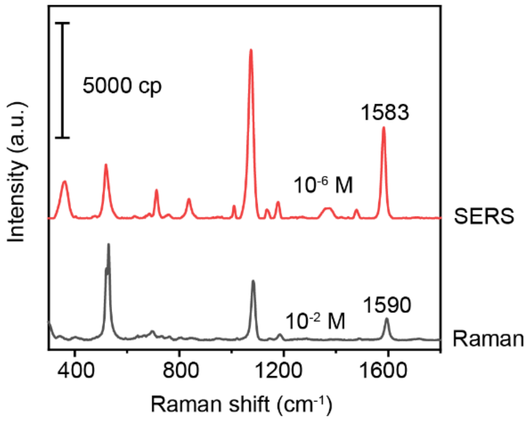

Figure S11 Normal Raman and SERS spectrum of 4-MBA.

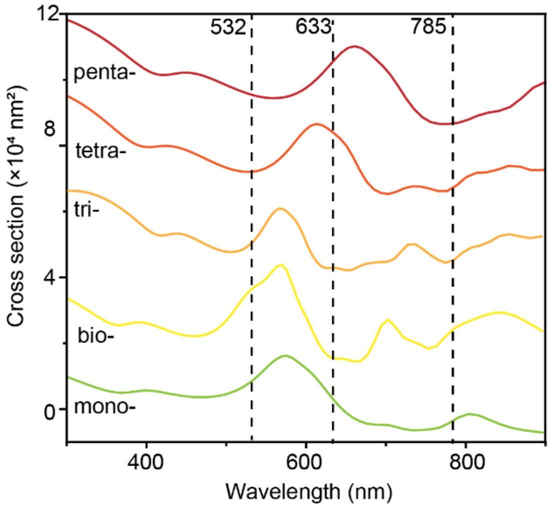

Figure S12 Calculated extinction cross-section spectra of mono-, bi-, tri-, tetra-, and penta-Au@Ag-Au NPs.

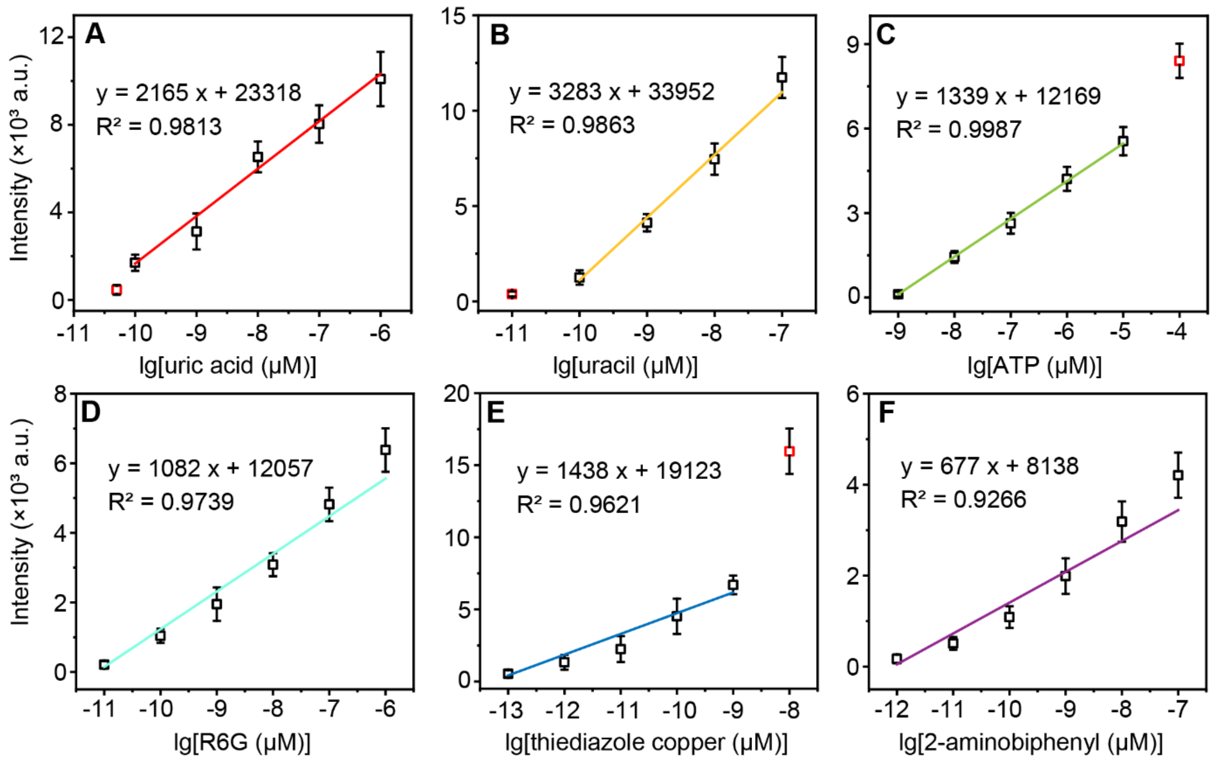

Figure S13 Raman peak intensity as a function of the logarithm concentrations of molecules (A-E: uric acid, uracil, adenosine triphosphate, rhodamine 6G, thiediazole copper, and 2-aminobiphenyl) collected from penta-Au@Ag-Au NPs.

Table S2 The Raman peak assignment of molecules used in this work.

Molecules	Peak position (cm^{-1})	Peak assignment
4-Mercaptobenzoic acid	1078/1583	Aromatic C–C ring stretching
Crystal violet	1625/1179	Aromatic C–C stretching/aromatic C–H bending
Rhodamine B	1650	C=C stretching
4-Aminothiophenol	1078	C−S and C-C ring stretching
rhodamine 6G	1513	Aromatic C–C stretching
Thiediazole copper	1378	C_5–N_4 stretching
2-Aminobiphenyl	1623	C=C stretching
Uric acid	642	Skeletal ring deformation
Uracil	791	Ring breathing
Adenosine triphosphate	829	Ring-breathing of adenine ring

Methylene blue	1621	C=C stretching

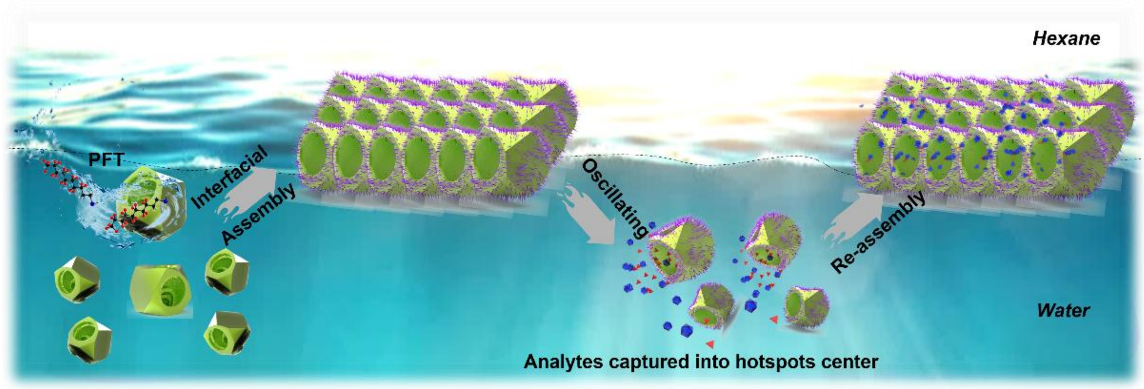

Figure S14 Schematic representation of the assembly of penta-Au@Ag-Au NPs accompanied by capturing analytes into effective hotspots at water-hexane interface.

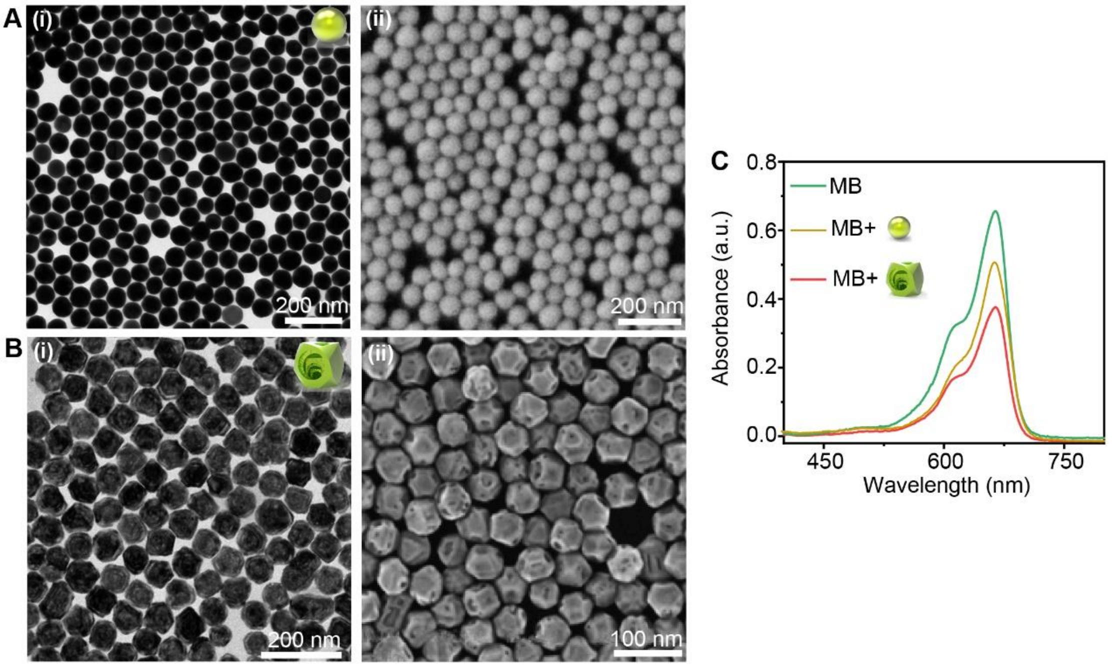

Figure S15 TEM (i) and SEM (ii) images of assembled AuNPs (A) and penta-Au@Ag-Au NPs (B) monolayer. (C) UV-vis spectra of MB before and after being adsorbed by two NPs monolayer.

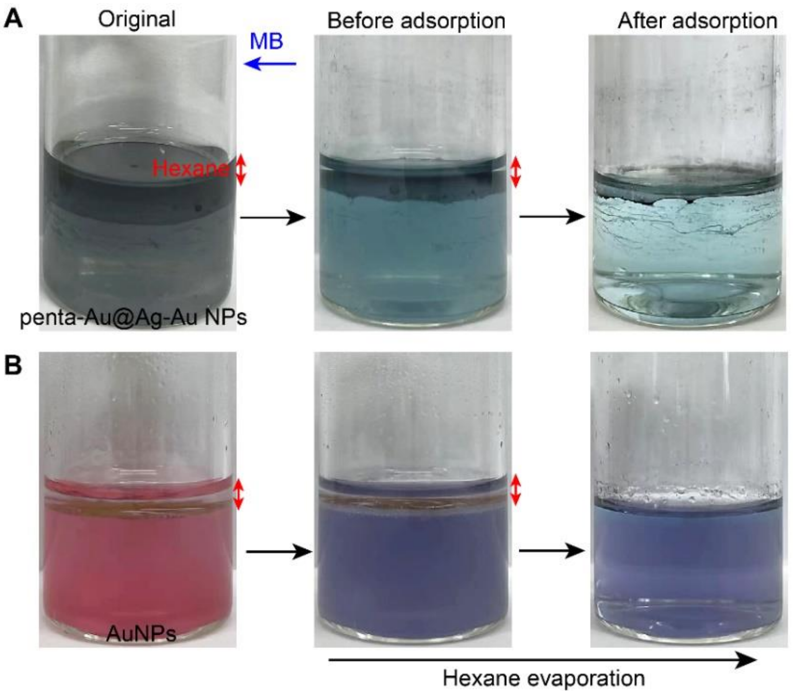

Figure S16 The photograph of assembled penta-Au@Ag-Au NPs (A) and AuNPs (B) monolayer used for capturing MB at the water-hexane interface.

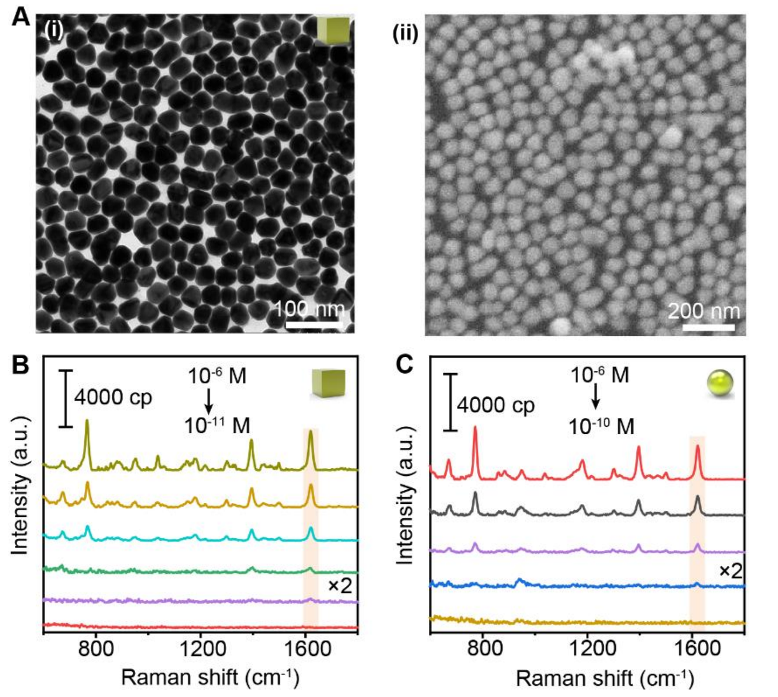

Figure S17 (A) The TEM (i) and SEM (ii) image of solid penta-Au@Ag-Au NPs monolayer. (B) SERS spectra of MB obtained from solid penta-Au@Ag-Au NPs and AuNPs monolayer.
